# Randomised controlled trial of weaning strategies for preterm infants on nasal continuous positive airway pressure

**DOI:** 10.1186/s12887-015-0462-0

**Published:** 2015-10-07

**Authors:** Jessica Tang, Shelley Reid, Tracey Lutz, Girvan Malcolm, Sue Oliver, David Andrew Osborn

**Affiliations:** University of Melbourne, Melbourne, Australia; RPA Newborn Care, Royal Prince Alfred Hospital, Missenden Road, Camperdown, Sydney, NSW 2050 Australia; Faculty of Nursing and Midwifery, University of Sydney, Sydney, NSW 2006 Australia; Discipline of Obstetrics, Gynaecology and Neonatology, University of Sydney, Sydney, NSW 2006 Australia

**Keywords:** High flow nasal cannula, Continuous positive airway pressure, Ventilator weaning, Infant, Premature

## Abstract

**Background:**

The optimal strategy for weaning very preterm infants from nasal continuous positive airway pressure (NCPAP) is unclear. Reported strategies include weaning NCPAP to a predefined pressure then trialling stopping completely (abrupt wean); alternate periods of increased time off NCPAP whilst reducing time on until the infant is completely weaned (gradual wean); and using high flow nasal cannula (HFNC) to assist the weaning process. The aim of this study was to determine the optimal weaning from NCPAP strategy for very preterm infants.

**Methods:**

A pilot single centre, factorial design, 4-arm randomised controlled trial. Sixty infants born <30 weeks gestation meeting stability criteria on NCPAP were randomly allocated to one of four groups. Group 1: abrupt wean with HFNC; Group 2: abrupt wean without HFNC; Group 3: gradual wean with HFNC; Group 4: gradual wean without HFNC. The primary outcomes were duration of respiratory support, chronic lung disease, length of hospital stay and time to full suck feeds.

**Results:**

The primary outcome measures were not significantly different between groups. Group 1 had a significant reduction in duration of NCPAP (group 1: median 1 day; group 2: 24 days; group 3: 15 days; group 4: 24 days; *p =* 0.002) and earlier corrected gestational age off NCPAP. There was a significant difference in rate of parental withdrawal from the study, with group 2 having the highest rate. Group 3 had a significantly increased duration on HFNC compared to group 1.

**Conclusions:**

Use of high flow nasal cannula may be effective at weaning infants from NCPAP but did not reduce duration of respiratory support or time to full suck feeds. Abrupt wean without the use of HFNC was associated with an increased rate of withdrawal by parent request.

**Trial registration:**

This study is registered at the Australian New Zealand Clinical Trials Registry (www.anzctr.org.au/). (Registration Number = ACTRN12610001003066).

## Background

Nasal continuous positive airway pressure (NCPAP) is effective at preventing intubation in preterm infants [1, 2] and preventing extubation failure in infants after mechanical ventilation [3]. Subsequently, various strategies have been trialled for the withdrawal of NCPAP in preterm infants [4]. Trials have compared a gradual reduction of NCPAP pressure versus increasing duration of time off; [5, 6] and also initially weaning pressure to 4-6cmH_2_O and then comparing attempts to take infants off NCPAP (‘abrupt weaning’) versus increasing duration of time off (‘gradual weaning’), with or without the addition of low flow nasal cannula [7]. This later study reported a decreased length of stay for babies randomised to a weaning strategy where NCPAP is simply stopped when infants met predefined stability criteria.

However, NCPAP has side effects including gaseous distension of the bowel, nasal trauma, and nasal deformity if NCPAP use is prolonged [8]. Heated, humidified high flow nasal cannula (HFNC) using flow rates greater than 1 L/min [9] are being used as an alternative to NCPAP. Surveys in Australia and the United Kingdom document its widespread use as an alternative to NCPAP, weaning off CPAP and post extubation [10, 11]. Trials comparing use of HFNC versus NCPAP for facilitating extubation in preterm infants report similar efficacy for prevention of extubation failure [12, 13] and reduced nasal trauma with HFNC [14]. Previous research reported that use of HFNC in preterm infants for weaning from NCPAP is associated with an increased exposure to oxygen and longer duration of respiratory support. [15] However, HFNC flow was restricted to 2 L/min and infants weaned from NCPAP were on a relatively high fraction inspired oxygen (FiO_2_ ≤ 0.3) so may have had relatively severe lung disease.

This is a pilot study designed to inform the optimal comparisons for a larger trial. The primary aim of a larger trial will be to determine the optimal method for weaning infants born <30 weeks gestation from NCPAP to reduce duration of respiratory support and time to full suck feeds. The secondary aims are to determine the efficacy of abrupt versus gradual weaning from NCPAP; and the efficacy of use of HFNC versus no HFNC for weaning infants from NCPAP.

## Methods

### Study population and study design

**Fig. 1:**
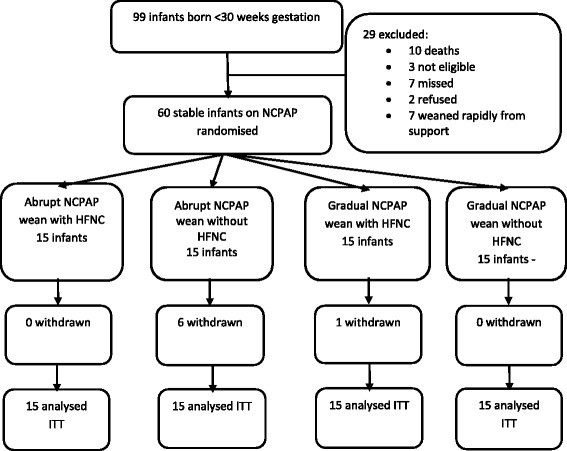
Flow Chart of the study showing patient allocation and follow up

This was a pilot, single-centre, prospective randomised control trial investigating the optimal method of weaning preterm infants from NCPAP using a 2 X 2 factorial design (Fig. 1) (ACTRN12610001003066). Informed parental consent was obtained before enrolment. Ethics approval for the study was obtained from the Sydney South West Area Health Service Human Ethics and Research Committee (X10-0262).

All infants born <30 weeks gestation on NCPAP at Royal Prince Alfred Hospital between October 2010 and June 2012 were eligible for inclusion in the study if they met the following criteria: 1) clinically stable on ≤5 cm H_2_O NCPAP (mouth closed); or 2) clinically stable on NCPAP (any level) but tolerating 6 h with mouth open; or 3) clinically stable on NCPAP (any level) and tolerating 6 h off NCPAP. Mouth closure was achieved by use of a chin strap or a pacifier and targeted to the infant’s work of breathing. A ≥6 FG gastric tube was used to avoid gastric over distension with air. Infants were excluded from study participation for the following reasons: 1) current infection with positive blood or CSF culture within previous 48 h; 2) major congenital or chromosomal abnormality; or 3) severe neurologic insult or neuromuscular disease.

### Intervention

Once informed parental consent was obtained, eligibility criteria [7] were confirmed by completing a randomisation form. Infants were randomised using sequentially numbered, opaque, sealed envelopes prepared in blocks of 4 to 8. The order of randomisation was allocated using a random number generator. Infants were randomised to one of four groups (Fig. 1):

- Group 1: Abrupt wean from NCPAP to HFNC. Infant was taken off NCPAP completely and put on HFNC starting at 6 L/min.
- Group 2: Abrupt wean from NCPAP without HFNC. Infant taken off NCPAP and received crib air or up to 25 % oxygen or low flow nasal cannula oxygen if required (≤1 L/min).
- Group 3: Gradual wean from NCPAP to HFNC. Infants gradually weaned off NCPAP by alternately placing onto HFNC for increasing lengths of time. As a guide, infants started at 6 h NCPAP and 1 h HFNC. Time on HFNC was increased by 1 h if stable, for each alternative period until 6 h on HFNC. Then NCPAP reduced by 1 h each alternative period until on continuous HFNC.
- Group 4: Gradual wean from NCPAP without HFNC. Infants gradually weaned off NCPAP by placing in crib air or up to 25 % oxygen or low flow nasal cannula oxygen if required (≤1 L/min) for increasing lengths of time. Infants started at 6 h NCPAP and 1 h off, with time off increased by 1 h if stable, each alternative period until off NCPAP. This was standard practice at RPA. Infants in groups 1 and 2 were placed back on NCPAP for at least 48 h or until stability criteria achieved if they met 2 or more failure criteria (derived from a previous trial [7]).

### Stability criteria

- NCPAP (mouth closed) ≤5 cm H_2_O,
- FiO_2_ ≤ 0.25 and not increasing,
- Respiratory rate ≤60 per minute,
- No significant chest recession,
- Less than 3 episodes of apnea, bradycardia, oxygen desaturation (<80 % for >20 s) in 1 h for the previous 12 h,
- Average oxygen saturation (SpO_2_) >86 % most of the time or PaO_2_ > 45 mmHg, and
- Not currently treated for patent ductus arteriosus (PDA) or sepsis.

### Failure criteria

- Increase work of breathing (intercostal recession and use of accessory muscles) with respiratory rate >75 per minute,
- Increased apnea and/or bradycardia and/or desaturations >2 in 1 h for the previous 6-h period,
- FiO_2_ requirement >0.25 to maintain SpO_2_ > 86 % and/or PaO_2_ > 45 mmHg,
- pH <7.2,
- PaCO_2_ > 65 mm Hg, or
- Apnea or bradycardia requiring resuscitation.

### Study devices

For HFNC, nasal cannula with outer diameter 2.4 mm (Fisher and Paykel Healthcare, Auckland, New Zealand) was connected to a circuit (Infant Oxygen Therapy System RT329, Fisher and Paykel) and humidifier (MR850, Fisher and Paykel). Flow rates were between 2 and 6 L/min. For NCPAP, short binasal prongs were used in conjunction with an underwater bubble NCPAP device (Fisher and Paykel) and flow rate was set ≥1 L/min above the ‘bubbling point’.

### Study outcomes

Primary outcomes were 1) chronic lung disease (CLD) defined as respiratory support or oxygen at 36 weeks’ corrected gestational age (cGA); 2) days respiratory support (NCPAP or HFNC or oxygen); 3) days of hospital stay; and 4) days to achieve full suck feeds. Secondary outcomes were 1) days NCPAP; 2) cGA off NCPAP; 3) HFNC days (from commencement); 4) pressure support days (NCPAP or HFNC); 5) cGA off pressure support; 6) cGA off respiratory support; 6) postnatal growth failure (weight <10th percentile) at 36 weeks cGA; 7) weight at 36 weeks’ cGA; 8) adverse events including grade 2 apnea (required intermittent positive pressure ventilation (IPPV)), pulmonary air leak, necrotising enterocolitis (NEC), PDA treatment, late onset sepsis; and 9) nasal injury. Outcomes are reported from time of randomisation unless otherwise specified.

### Statistical analysis

All data were analysed using SPSS (IBM SPSS Statistics version 21.0) using 2-sided tests and intention to treat (ITT) analysis. The data for infants withdrawn from treatment is reported in group of assignment. Primary analysis is reported for the 4 groups. In view of the factorial design, a secondary analysis is reported for combined groups: abrupt wean versus gradual wean; and HFNC versus no HFNC. All analyses were prespecified in the protocol. Dichotomous data are reported as medians and interquartile range (IQR) or means and standard deviation (sd) where appropriate. As a substantial proportion of time-related data had skewed distributions, non-parametric statistics were predominately reported. Statistical significance was assessed using ANOVA and Student t-test for differences in means of parametric data, and independent sample Kruskal-Wallis and Mann–Whitney U tests for non-parametric data. Dichotomous data were analysed using Pearson chi [2] or Fisher exact test where appropriate. Statistical significance was assumed at the *p* ≤ 0.05 level for primary outcomes and *p* ≤ 0.01 for secondary outcomes. Sample size calculation was not performed as this was a pilot study.

## Results

Ninety infants were born <30 weeks gestational age October 2010 and June 2012. Sixty eligible infants were enrolled and randomised, 15 to each group. Reasons for non-enrolment are reported in Fig. 1. All infants received the allocated treatment and were analysed by intention to treat. The groups were well balanced for perinatal and clinical characteristics after randomisation (Table 1). Infants randomised had a mean gestation 27.5 weeks (range 24.0–29.9) and birth weight 989 g (574–1617). They were aged 28 days (range 2–76) with mean postmenstrual age 31 weeks (27–37) and weight 1237 g (662–1890) and were similar between groups. Infants were on mean FiO_2_ 0.21 (range 21–23), pressure 5 cmH_2_0 (5–5), on NCPAP for 19 h (5–24) and tolerated 5 h (0–15) off NCPAP and were similar between groups.

**Table 1:** Baseline perinatal and clinical characteristics of groups at randomisation **(**n (%) or median (IQR) unless specified)

	Group 1	Group 2	Group 3	Group 4	*p*
	(*n =* 15)	*n =* 15	*n =* 15	*n =* 15	
Mean gestation (sd)	27.7 (1.5)	27.1 (1.8)	27.5 (1.3)	27.7 (1.1)	0.6
Mean birthweight - g (sd)	1027 (229)	945 (211)	975 (280)	1010 (282)	0.8
Complete corticosteroids	12 (80 %)	9 (60 %)	8 (53 %)	13 (87 %)	0.1
Mother in labour	7 (47 %)	6 (40 %)	7 (47 %)	6 (40 %)	0.9
Caesarean	14 (93 %)	11 (73 %)	12 (80 %)	12 (80 %)	0.5
Chorioamnionitis	6 (40 %)	4 (27 %)	4 (27 %)	4 (27 %)	0.6
Male	4 (26 %)	5 (33 %)	9 (60 %)	7 (47 %)	0.3
Mechanical ventilation	15 (100 %)	14 (93 %)	14 (93 %)	13 (87 %)	0.5
Surfactant	14 (93 %)	13 (87 %)	14 (93 %)	14 (93 %)	0.9
Diuretics	5 (33 %)	2 (13 %)	4 (27 %)	2 (13 %)	0.4
Grade 2 apnea (required IPPV)	1 (7 %)	2 (13 %)	3 (20 %)	2 (13 %)	0.8
Caffeine	15 (100 %)	15 (100 %)	15 (100 %)	15 (100 %)	
Full enteral feeds	10 (67 %)	10 (67 %)	9 (60 %)	8 (53 %)	0.9
NEC	1 (7 %)	1 (7 %)	1 (7 %)	0	0.5
Treated ductus arteriosus	5 (33 %)	10 (67 %)	7 (47 %)	4 (27 %)	0.1
Intraventricular haemorrhage	5 (33 %)	3 (20 %)	4 (27 %)	1 (7 %)	0.5
Late onset sepsis	1 (7 %)	4 (27 %)	4 27(%)	5 (33 %)	0.3
Nasal trauma	1 (7 %)	1 (7 %)	0 (0 %)	1 (7 %)	0.4
Corrected gestation	30.3	30.6	32.1	30.0	0.3
	(29.4, 33.0)	(29.4, 32.1)	(29.9, 34.7)	(27.9, 32.6)	
Mean weight - g (sd)	1218 (170)	1253 (294)	1342 (312)	1139 (318)	0.3

Seven infants were withdrawn at parent request from the allocated treatment, 6 (40 %) infants who were allocated to group 2 (abrupt NCPAP wean without HFNC) and 1 infant allocated to group 3 (gradual NCPAP wean with HFNC). The difference in withdrawal rate was statistically significant (ANOVA *p =* 0.01). The reason for withdrawal of all infants was dissatisfaction with weaning method. Infant outcomes are reported for all infants in an intention to treat analysis.

### Four-group comparison

No significant difference was found between groups for primary outcomes including CLD, respiratory support days, days to full suck feeds and days of hospital stay from randomisation (Table 2). There was a significant difference in duration of NCPAP between groups with group 1 (abrupt wean with HFNC) having a median 1 day on NCPAP, compared to group 2 with 24 days, group 3 with 15 days and group 4 with 24 days (ANOVA *p =* 0.002). Group 1 had a significantly reduced duration of NCPAP and cGA off NCPAP compared to groups 2–4 combined (Fisher exact test p < 0.01). There was a significant difference between groups 1 and 3 in days HFNC from start of treatment (median 15 days versus 30 days; *p =* 0.004). There were no significant differences between groups in days of pressure support, cGA off pressure support, cGA off respiratory support, cGA at full suck feeds, cGA at hospital discharge and days of caffeine use. Incidences of adverse events (grade 2 apnea, NEC, PDA treatment, ROP and laser treatment) after randomisation were not significantly different. No infant was diagnosed with periventricular leucomalacia or had a PDA ligation.

**Table 2:** Infant outcomes of four groups (data from randomisation; n (%) or median (IQR) unless specified)

	Group 1	Group 2	Group 3	Group 4	ANOVA
	*n =* 15	*n =* 15	*n =* 15	*n =* 15	*p*-value
CLD at 36 weeks	3 (20 %)	4 (27 %)	7 (47 %)	2 (13 %)	0.2
Days respiratory support	21 (9, 33)	26 (20, 38)	30 (24, 33)	24 (10, 35)	0.4
Days hospital stay	50 (39, 58)	53 (41, 71)	64 (50, 78)	53 (48, 66)	0.2
Days to full suck feeds	40 (35, 54)	51 (36, 66)	57 (41, 73)	51 (37, 64)	0.5
Days NCPAP	1 (0, 12)	24 (9, 28)	15 (11, 21)	24 (10, 35)	0.002
Gestational age off NCPAP	31.6	33.9	35.7	34.6	0.04
	(30.0, 34.1)	(32.1, 35.9)	(31.0, 37.6)	(31.9, 35.3)	
Days HFNC from start of treatment	15 (7, 24)		30 (20, 34)		0.004
Days pressure support	15 (9, 29)	24 (9, 28)	30 (24, 33)	24 (10, 35)	0.1
Gestational age off pressure support	34.0	33.9	35.9	34.6	0.07
	(32.7, 35.3)	(32.1, 35.9)	(33.9, 38.9)	(31.9, 35.3)	
Gestational age off respiratory support	34.7	34.1	35.9	34.6	0.1
	(33.4, 35.3)	(33.1, 36.0)	(33.9, 38.9)	(31.9, 35.3)	
Gestational age at full suck feeds	36.9	37.1	39.6	37.3	0.1
	(36.4, 38.0)	(36.1, 40.6)	(37.1, 44.0)	(36.6, 39.1)	
Gestation at discharge	37.7	37.9	39.9	38.3	0.1
	(36.9, 39.1)	(37.1, 40.1)	(37.9, 45.0)	(36.9, 39.7)	
Postnatal growth failure	9 (60 %)	9 (60 %)	7 (47 %)	7 (47 %)	0.8
Weight at 36 weeks - g (sd)	2158 (411)	2044 (390)	2042 (338)	2128 (532)	0.4
Days caffeine	24 (24)	34 (30)	40 (22)	39 (33)	0.2
Grade 2 apnea (required IPPV)	0	1 (7 %)	0	0	0.4
Necrotising enterocolitis	0	1 (7 %)	0	0	0.4
Treated ductus arteriosus	2 (13 %)	5 (33 %)	4 (27 %)	1 (7 %)	0.2
Retinopathy of prematurity	7 (47 %)	6 (40 %)	6 (40 %)	6 (40 %)	0.6
Laser therapy	1 (7 %)	0	0	1 (7 %)	0.6
Nasal injury	0	0	1 (7 %)	1 (7 %)	0.4
Withdrawn	0	6 (40 %)	1 (7 %)	0	0.01

### Combined groups: HFNC versus no HFNC

No significant difference was found in primary outcomes between infants receiving HFNC versus no HFNC (Table 3). Infants allocated HFNC had a significant reduction in duration of NCPAP (median 12 days versus 24 days; *p =* 0.009). There were no significant differences in days of pressure support, cGA off pressure support, cGA off respiratory support, cGA at full suck feeds and cGA at hospital discharge.

**Table 3:** Outcomes of combined HFNC groups versus no HFNC groups (data from randomisation; n (%) or median (IQR) unless otherwise specified)

	HFNC	No HFNC	*p*-value
	*n =* 30	*n =* 30	
CLD at 36 weeks	10 (33 %)	6 (20 %)	0.2
Days respiratory support	28 (10, 36)	24 (16, 33)	0.7
Days hospital stay	56 (42, 67)	53 (46, 68)	0.7
Days to full suck feeds	47 (36, 65)	51 (39, 62)	0.5
Days NCPAP	12 (10, 33)	24 (1, 17)	0.009
Gestational age off NCPAP	33.0 (32.1, 35.4)	33.9 (30.9, 35.7)	0.4
Days HFNC from start of treatment	24 (11, 32)	*	
Days pressure support	27 (10, 33)	24 (15, 33)	0.3
Gestational age off pressure support	34.9 (32.1, 35.4)	33.9 (33.4, 36.8)	0.09
Gestational age off respiratory support	35.0 (32.5, 35.6)	34.4 (33.5, 36.8)	0.1
Gestational age at full suck feeds	37.6 (36.4, 39.2)	37.3 (36.7, 39.9)	0.5
Gestational age at discharge	38.4 (37.1, 39.7)	38.1 (37.3, 41.4)	0.3

* not applicable

### Combined groups: abrupt wean versus gradual wean

No significant difference in primary outcomes was found between infants allocated abrupt wean versus gradual wean (Table 4). Infants allocated abrupt wean had a significant reduction in duration of HFNC (median 15 days versus 30 days; *p =* 0.003). There were no significant differences in other secondary outcomes at the prespecified level (*p* ≤ 0.01). However, infants allocated abrupt wean had fewer days NCPAP (10.5 days versus 16.5 days; *p =* 0.02), reduced cGA off NCPAP (33.1 weeks versus 34.6 weeks; *p =* 0.05), and fewer days pressure support (21.5 days versus 27.5 days; *p =* 0.04).

**Table 4:** Outcomes of combined abrupt versus gradual NCPAP wean groups (data from randomisation; n (%) or median (IQR) unless specified)

	Abrupt NCPAP wean	Gradual NCPAP wean	*p*-value
	*n =* 30	*n =* 30	
CLD at 36 weeks	7 (23 %)	9 (30 %)	0.6
Days respiratory support	24 (13, 34)	28 (17, 34)	0.4
Days hospital stay	52 (41, 63)	61 (48, 69)	0.1
Days to full suck feeds	46.5 (35, 58)	54.5 (41, 65)	0.2
Days NCPAP	11 (1, 26)	17 (10, 29)	0.02
Gestational age off NCPAP	33.1 (31.0, 34.6)	34.6 (31.8, 36.1)	0.05
Days HFNC from start of treatment	15 (7, 24)	30 (20, 34)	0.003
Days pressure support	22 (9, 28)	28 (17, 34)	0.04
Gestational age off pressure support	33.9 (32.6, 35.4)	34.9 (32.7, 36.7)	0.2
Gestational age off respiratory support	34.4 (33.1, 35.9)	34.9 (32.7, 36.7)	0.7
Gestational age at full suck feeds	37.1 (36.4, 39.1)	38.7 (36.8, 39.9)	0.1
Gestational age at discharge	37.8 (37.1, 39.4)	38.7 (37.4, 41.0)	0.2

## Discussion

This study was a pilot designed to determine the optimal comparisons for a larger trial. None of the strategies resulted in a significant effect on the prespecified primary outcomes including incidence of CLD, duration of respiratory support, days to full suck feeds or hospital stay although the study is underpowered to find a difference. However, there were significant differences between groups in days of NCPAP and infants withdrawn from treatment due to parental concern. The group abruptly weaning infants to HFNC had the shortest duration of NCPAP. The group abruptly weaned without use of HFNC had the highest withdrawal rate. In combined group analysis, infants on HFNC had a significant reduction in days NCPAP. Use of HFNC may be an efficient method for weaning infants from NCPAP even though it did not reduce the overall duration of respiratory support, days to full suck feeds or duration of hospital stay. In combined group analysis, abruptly weaning infants reduced the duration of HFNC required. This suggests the best strategy for weaning infants from NCPAP is to place them on HFNC when they are at a predefined level of pressure support. Although abrupt weaning was also associated with a reduced duration of NCPAP, corrected gestational age off NCPAP and duration of pressure support, this did not reach our predefined significance level for secondary outcomes.

HFNC delivers continuous distending pressure [16]. The delivered continuous distending pressure is higher in smaller infants (<1500 g) [17], at higher flow rates [17–20], using prongs with a larger outer diameter [19], and when the infant’s mouth is closed [19]. Previous research that assessed use of HFNC in preterm infants for weaning from NCPAP reported use of HFNC was associated with an increased exposure to oxygen and longer duration of respiratory support. [15] However, in that study HFNC flow used prongs with an outer diameter of 0.3 cm and flow was restricted to 2 L per minute. In addition, infants were weaned from NCPAP when on a relatively high fraction inspired oxygen (≤0.3) suggesting the infants had more severe lung disease and were on a higher level of respiratory support. In contrast, our study weaned infants on NCPAP at 5cmH_2_O, the majority of whom were in air, and used HFNC with an outer diameter of 0.2 cm and commenced at 6 L/min. The efficiency of HFNC in this study may be due to the use of higher flow rates for weaning infants from lower levels of respiratory support.

Two recent trials comparing use of HFNC versus NCPAP for facilitating extubation in preterm infants report similar efficacy for prevention of extubation failure [12, 13] and reduced nasal trauma with HFNC [14]. It is noteworthy that these trials did not report routine mouth closure techniques for infants allocated NCPAP. Mouth open is associated with loss of pharyngeal pressure support and potentially efficacy of NCPAP [21]. A third trial comparing HFNC versus NCPAP applied immediately post extubation or early as initial non-invasive support for respiratory dysfunction, reported similar efficacy including no difference in early failure or need for intubation [22]. Infants on HFNC had an increased duration of pressure support although there was no difference in duration of oxygen, bronchopulmonary dysplasia or duration of hospitalisation. These trials and the current study suggest HFNC has similar efficacy to NCPAP for infants in need of lower levels of respiratory support. A previous trial that assessed a practice of abrupt weaning versus gradual weaning from NCPAP when infants met prespecified stability criteria, reported that abrupt weaning from NCPAP was associated with a shorter duration of oxygen and time on respiratory support. [7] However, the trial had substantial differences in baseline characteristics including gender and condition at birth suggesting the results should be treated with caution. Our trial had a similar set of ‘stability’ and ‘failure’ criteria. However, abrupt weaning without HFNC was associated with a significantly increased rate of parental withdrawal and no significant benefits. The reason for withdrawal of all infants was dissatisfaction with weaning method. Parents reported feeling their infant was ‘failing the weaning process’ when attempting to abruptly cease NCPAP. The analyses from our trial suggest a strategy of abrupt wean with use of HFNC may be the most efficient and acceptable to parents. Given this is a small pilot study caution is advised in interpreting the findings.

Given HFNC has been demonstrated to reduce nasal trauma [14, 22], a trial of abrupt weaning of NCPAP with HFNC versus gradual weaning of NCPAP may be difficult to justify for infants on lower level respiratory support. Further research is required to further define the role of HFNC for primary respiratory support of newborn infants and infants being extubated from mechanical ventilation.

## Conclusion

Use of high flow nasal cannula was effective at weaning infants from NCPAP. Further trials are required to determine if use of HFNC for weaning can reduce the duration of pressure support or reduce time to full suck feeds. A strategy of weaning NCPAP to a predefined level and then stopping NCPAP completely without use of high flow nasal cannula was associated with increased rate of withdrawal at parent request so may not be acceptable in all settings.

## Abbreviations

cGA: Corrected gestational age
CLD: Chronic lung disease
FiO_2_: Inspired concentration of oxygen
HFNC: High flow nasal cannula
IQR: Interquartile range
NCPAP: Nasal continuous positive airway pressure
NEC: Necrotising enterocolitis
PDA: Patent ductus arteriosus
ROP: Retinopathy of prematurity
